# Multiple Aneurysms AnaTomy CHallenge 2018 (MATCH): uncertainty quantification of geometric rupture risk parameters

**DOI:** 10.1186/s12938-019-0657-y

**Published:** 2019-03-25

**Authors:** Leonid Goubergrits, Florian Hellmeier, Jan Bruening, Andreas Spuler, Hans-Christian Hege, Samuel Voss, Gábor Janiga, Sylvia Saalfeld, Oliver Beuing, Philipp Berg

**Affiliations:** 1 Institute for Computational and Imaging Science in Cardiovascular Medicine, Charité-Universitätsmedizin Berlin, Augustenburger Platz 1, 13353 Berlin, Germany; 2 Helios Hospital Berlin Buch, Berlin, Germany; 3 Zuse Institute, Berlin, Germany; 4 Department of Fluid Dynamics and Technical Flows, University of Magdeburg, Magdeburg, Germany; 5 Department of Simulation and Graphics, University of Magdeburg, Magdeburg, Germany; 6 Institute of Neuroradiology, University Hospital Magdeburg, Magdeburg, Germany; 7 Forschungscampus STIMULATE, Magdeburg, Germany

**Keywords:** Uncertainty, Cerebral aneurysms, Segmentation, Rupture risk

## Abstract

**Background:**

Geometric parameters have been proposed for prediction of cerebral aneurysm rupture risk. Predicting the rupture risk for incidentally detected unruptured aneurysms could help clinicians in their treatment decision. However, assessment of geometric parameters depends on several factors, including the spatial resolution of the imaging modality used and the chosen reconstruction procedure. The aim of this study was to investigate the uncertainty of a variety of previously proposed geometric parameters for rupture risk assessment, caused by variability of reconstruction procedures.

**Materials:**

26 research groups provided segmentations and surface reconstructions of five cerebral aneurysms as part of the Multiple Aneurysms AnaTomy CHallenge (MATCH) 2018. 40 dimensional and non-dimensional geometric parameters, describing aneurysm size, neck size, and irregularity of aneurysm shape, were computed. The medians as well as the absolute and relative uncertainties of the parameters were calculated. Additionally, linear regression analysis was performed on the absolute uncertainties and the median parameter values.

**Results:**

A large variability of relative uncertainties in the range between 3.9 and 179.8% was found. Linear regression analysis indicates that some parameters capture similar geometric aspects. The lowest uncertainties < 6% were found for the non-dimensional parameters isoperimetric ratio, convexity ratio, and ellipticity index. Uncertainty of 2D and 3D size parameters was significantly higher than uncertainty of 1D parameters. The most extreme uncertainties > 80% were found for some curvature parameters.

**Conclusions:**

Uncertainty analysis is essential on the road to clinical translation and use of rupture risk prediction models. Uncertainty quantification of geometric rupture risk parameters provided by this study may help support development of future rupture risk prediction models.

**Electronic supplementary material:**

The online version of this article (10.1186/s12938-019-0657-y) contains supplementary material, which is available to authorized users.

## Background

Intracranial aneurysms (IAs) are complexly shaped local dilatations of the cerebral vasculature. They occur at different locations in the anterior and posterior cerebral circulation and are unique in size and phenotype [1, 2]. Unruptured IAs are found in approximately 3.2% of the adult population worldwide [3] and are being discovered with an increasing frequency due to the widespread use of high-resolution magnetic resonance imaging or computed tomography [3].

However, according to published data of two large studies (the International Study of Unruptured Intracranial Aneurysms (ISUIA) [4] and a Japanese study of unruptured aneurysms [5]), the large majority of IAs never rupture. As aneurysm treatment is associated with significant risks, physicians have to weigh the risk of treatment against the rupture risk of yet unruptured aneurysms.

Rupture risk of unruptured IAs increases with increasing aneurysmal diameter [4] and current clinical guidelines therefore recommend to treat aneurysms larger than 7 mm, it was shown that smaller aneurysms can also lead to subarachnoid hemorrhage [6, 7]. To integrate other clinical factors for the assessment of IA rupture risk, (e.g., specific population, arterial hypertension, age, sex, previous rupture of an independent intracranial aneurysm, site), the PHASES score was developed [8, 9]. However, it has been demonstrated that in practice PHASES underestimates individual rupture risk and may lead to an inappropriate treatment [10, 11].

Therefore, advanced techniques were developed to improve assessment of aneurysm morphology. Raghavan et al. [12] defined several metrics (e.g., nonsphericity index, undulation index, ellipticity index) to enable accurate description of complex aneurysm shapes. In the last decade, multiple geometric parameters were added [13–16]. Nevertheless, none of these complex quantifications are introduced into clinics yet. Physicians still prefer assessment of rupture risk using one-dimensional or normalized features such as aneurysm diameter or aspect ratio. One reason for this is, that these parameters can be measured directly from imaging data, while complex geometric risk parameters require segmentation and reconstruction of the aneurysm’s 3D geometry.

To provide a clinically applicable workflow, Saalfeld et al. [17] analysed 100 IAs by means of a semi-automatic neck curve reconstruction algorithm. The authors identified the characteristic dome point angle as a potential morphologic candidate to assess rupture risk. A very recent literature review by Liang et al. [18], included 46 studies containing 2791 aneurysms and found the simple metrics aspect ratio and aneurysm size as the most relevant parameters for rupture prediction. As these studies depend strongly on specific processing techniques, inter-study comparability might not be given concerning the lack of a gold standard for segmentation. Multiple algorithms are available for general vessel segmentation, but are not specifically tailored to the reconstruction of IAs [19]. Furthermore, custom-made smoothing algorithms, the subjective impression of the corresponding investigator, and different levels of experience might result in further inaccuracies.

Models for discriminating between ruptured and unruptured aneurysms proposed for a cohort available to one research group often fail when applied to other patient cohorts. While the segmentation procedure might be standardized fairly well in one group, other research groups might use different methods as no consensus on image segmentation of cerebral aneurysms exists. Uncertainties caused by different image segmentation methods will result in uncertainties in geometric and hemodynamic parameters used for rupture risk prediction. These uncertainties are major factors preventing successful introduction of image-based methods in clinical practice [20, 21].

Accuracy of segmentation and the impact of its uncertainties were investigated by several studies. Berg et al. investigated the impact of voxel size and reconstruction kernel on morphologic and hemodynamic parameters. They found a higher susceptibility of parameters associated with neck representation and wall shear stress [22, 23]. Klepaczko et al. found a variation of vessel size between 7 and 79% depending on the vessel size, image acquisition and segmentation method [24]. However, most studies focus on the impact of segmentation on hemodynamic parameters [25–28]. As of yet, no information is available about uncertainties of morphometric rupture risk parameters caused by inter-group variability in segmentation.

Therefore, the international Multiple Aneurysms AnaTomy CHallenge (MATCH) was announced to directly compare segmentation capabilities based on identical aneurysm datasets. Within MATCH, 26 groups from 13 countries contributed the segmentation results of five IAs with different location, size, and shape. Initial results revealed that remarkable differences with respect to vessel and aneurysm representation occurred [29].

To further address this situation, the current study is a follow-up analysis of reconstructed IAs contributed within MATCH. The aim is to quantify the segmentation-related uncertainty of multiple geometric parameters previously proposed for rupture risk prediction. This analysis of variability is intended to enable physicians and biomedical engineers to critically assess those quantities. Finally, the knowledge of segmentation uncertainty could affect the performance of rupture risk prediction models both directly and indirectly. This is because geometric parameters and hemodynamic parameters (based on computational fluid dynamics simulations), both of which are affected by geometry reconstruction, have been proposed as rupture risk predictors [3, 12, 30, 31]. Furthermore, parameters with low uncertainty can be identified for preferential use in future studies.

## Methods

In order to quantify the uncertainties of a set of geometric parameters proposed in the literature as possible rupture risk predictors, reconstructions of five IAs provided in the MATCH challenge were analyzed.

### Multiple Aneurysms AnaTomy CHallenge (MATCH)

The international challenge was announced on November 3rd, 2017. Interested groups were provided with anonymized clinical DICOM datasets, acquired with an interventional angio suite (Artis Q, Siemens Healthineers, Forchheim, Germany). Rotational angiographies were extracted within a 5 s run comprising 133 slices with a voxel resolution of 1240 × 960. From the rotational angiographies, a 3D digital subtraction angiography CT scan (512 × 512 × 512) was reconstructed exhibiting an isotropic voxel size of 0.28 mm. The datasets comprised three 3D digital subtraction angiography volumes containing the left anterior, right anterior, and posterior cerebral circulation. In total, the corresponding patient harbored five IAs: three in the left and right M1-segment of the middle cerebral artery (MCA), one on the left MCA bifurcation, and one on the left posterior inferior cerebellar artery.

To assess the real-world variability of existing segmentation techniques, each team was requested to apply their own algorithms and strategies. Research groups were allowed to submit their contributions until the end of January 2018 and in total 26 research teams from 13 countries participated. However, group 5 had to be excluded completely.

Overall, 18 different software tools were used for segmentation. Furthermore, four different segmentation algorithms were applied and large variation with respect to the required processing time was reported. For further details regarding the design and initial outcome of MATCH, please refer to Berg et al. [29].

### Geometric rupture risk parameters

Altogether, 40 geometric parameters were calculated. These comprise twelve aneurysm size (#1–12), six neck size (#13–18), twelve non-dimensional (#19–30), as well as ten curvature parameters (#31–40), respectively. Table 1 summarizes the corresponding geometric rupture risk parameters, including a short description and references to the definitions used.

**Table 1:** Complete list of all geometric parameters

**#**	Name (abbreviation), unit [reference]	Short description
1	Height (H), mm [34]	Maximum perpendicular distance from the neck plane to the aneurysm surface
2	Maximum dimension (L_max_), mm [40]	Maximum distance between two points on the aneurysm surface
3	Maximum height (H_max_), mm [14]	Maximum distance between the centroid of the neck and the aneurysm surface
4	Maximum diameter (D_max_), mm	Maximum neck plane-parallel distance between two points on the aneurysm surface
5	Bulge height (H_b_), mm [34, modified]	Distance between the neck plane and the maximum diameter
6	Surface area (A), mm^2^ [14]	Surface area of the aneurysm
7	Convex hull surface area (A_CH_), mm^2^ [14]	Surface area of the aneurysm’s convex hull
8	Minimal bounding sphere surface area (A_MBS_), mm^2^ [41]	Surface area of the aneurysm’s minimal bounding sphere
9	Closed surface area (A_closed_), mm^2^	Surface area of the neck-closed aneurysm
10	Volume (V), mm^3^ [14]	Volume of the neck-closed aneurysm
11	Convex hull volume (V_CH_), mm^3^ [14]	Volume of the aneurysm’s convex hull
12	Minimal bounding sphere volume (A_MBS_), mm^3^ [41]	Volume of the aneurysm’s minimal bounding sphere
13	Minimum neck diameter (D_neck,min_), mm	Minimum distance between two opposite points on the neck perimeter
14	Maximum neck diameter (D_neck,max_), mm [42]	Maximum distance between two points on the neck perimeter
15	Neck perimeter (P_neck_), mm [34, modified]	Perimeter of the aneurysm neck
16	Equivalent neck diameter (D_neck,equiv_), mm [34, modified]	Hydraulic diameter of the aneurysm neck
17	Neck area (A_neck_), mm^2^ [34, modified]	Area of the aneurysm neck
18	Elliptical neck area (A_neck,elliptical_), mm^2^	Neck area calculated by using the product of the minimum and maximum neck diameter
19	Size ratio (SR) [14]	Ratio of aneurysm size to the parent vessel diameter
20	Aspect ratio (AR) [42, modified]	Ratio of height to maximum neck diameter
21	Equivalent aspect ratio (eAR) [34, modified]	Ratio of height to equivalent neck diameter
22	Bottleneck factor (BF) [34, modified]	Ratio of maximum diameter to maximum neck diameter
23	Bulge location (BL) [34, modified]	Ratio of bulge height to height
24	Nonsphericity index (NSI) [14]	Normalized ratio of volume to surface area relative to a hemisphere
25	Isoperimetric ratio (IPR) [34]	Normalized ratio of surface area to volume
26	Aneurysm volume to bounding sphere volume (AVSV) [41]	Ratio of volume to minimal bounding sphere volume
27	Aneurysm surface area to bounding sphere area (AASA) [41]	Ratio of surface area to minimal bounding sphere surface area
28	Undulation index (UI) [14]	1 minus the ratio of volume to convex hull volume
29	Convexity ratio (CR) [34]	Ratio of volume to convex hull volume
30	Ellipticity index (EI) [14]	Normalized ratio of convex hull volume to convex hull surface area relative to a hemisphere
31	Mean of mean curvature (MAA), mm^−1^ [34]	Surface average of local mean curvature
32	Mean of absolute mean curvature (absMAA), mm^−1^	Surface average of the magnitude of the local mean curvature
33	Standard deviation of mean curvature (MSD), mm^−1^	Standard deviation of the local mean curvature
34	High mean curvature (HMC), % [41, modified]	Relative increase of the mean of absolute mean curvature over the mean curvature of the minimal bounding sphere, in percent
35	L2-norm of mean curvature (MLN) [34]	Scale invariant measure of surface irregularity, uses mean curvature
36	Mean of Gaussian curvature (GAA), mm^−2^ [34]	Surface average of local Gaussian curvature
37	Mean of absolute Gaussian curvature (absGAA), mm^−2^	Surface average of the magnitude of the local Gaussian curvature
38	Standard deviation of Gaussian curvature (GSD), mm^−2^	Standard deviation of the local Gaussian curvature
39	High Gaussian curvature (HGC), % [41, modified]	Relative increase of the mean of absolute Gaussian curvature over the Gaussian curvature of the minimal bounding sphere, in percent
40	L2-norm of Gaussian curvature (GLN) [34]	Scale invariant measure of surface irregularity, uses Gaussian curvature

List of all geometric parameters that were calculated for the MATCH dataset. The parameter names, abbreviations, units, and the original reference are specified. The last column provides a short description of the parameters

Note that this set of parameters does not represent all geometric parameters previously proposed in literature. For example, we excluded parameters whose definition requires manual, subjective processing steps (e.g. [32]), as well as parameters whose description in the literature did not allow us to accurately reproduce parameter calculation (e.g. [33]).

### Calculation of parameters

Prior to parameter calculation, all five aneurysms were manually extracted from the segmentations provided by the 26 research groups participating in the challenge. Extraction was done using ZIBAmira (v. 2015.28, Zuse Institute Berlin, Germany). Since not every research group segmented every aneurysm, this resulted in a total of 121 aneurysm geometries. The aneurysm geometries were then checked for manifoldness, self-intersections, consistent triangle orientation, and lack of holes in the aneurysm surface and any such geometric errors were manually corrected.

Subsequently, the geometric parameters were automatically calculated for all aneurysm geometries using ZIBAmira, MATLAB (R2017b, MathWorks, Natick, USA), and Python (v. 3.7.1, Python Software Foundation, Delaware, USA) scripts.

All final aneurysm geometries as well as the table containing all parameter values for all aneurysms are provided as supplemental material, see “Availability of data and materials” section as well as Additional file 1.

### Uncertainty quantification

First, median and the middle 68.3% range (i.e. the difference between the 84.13th and the 15.87th percentile) were calculated for each geometric parameter and aneurysm using SPSS (v. 23, IBM, Armonk, USA). This absolute uncertainty range corresponds to the range of mean ± one standard deviation for normally distributed data. We chose this range to allow comparison of reported uncertainty ranges against standard deviations, which are commonly reported for normally distributed data. The relative uncertainty of a parameter was then defined as the uncertainty range divided by the median value, in percent. Thus, for each parameter five relative uncertainties were calculated, one for each aneurysm.

Finally, these relative uncertainties were averaged across all five aneurysms. The average and variation of the relative uncertainty for all aneurysms were then specified using the median and the interquartile range (IQR), respectively. We decided to specify the IQR rather than the range, as the range is more sensitive to outliers. Additionally, linear regression between the absolute uncertainty range (as defined above) and the median calculated for each aneurysm was investigated.

**Fig. 1:**
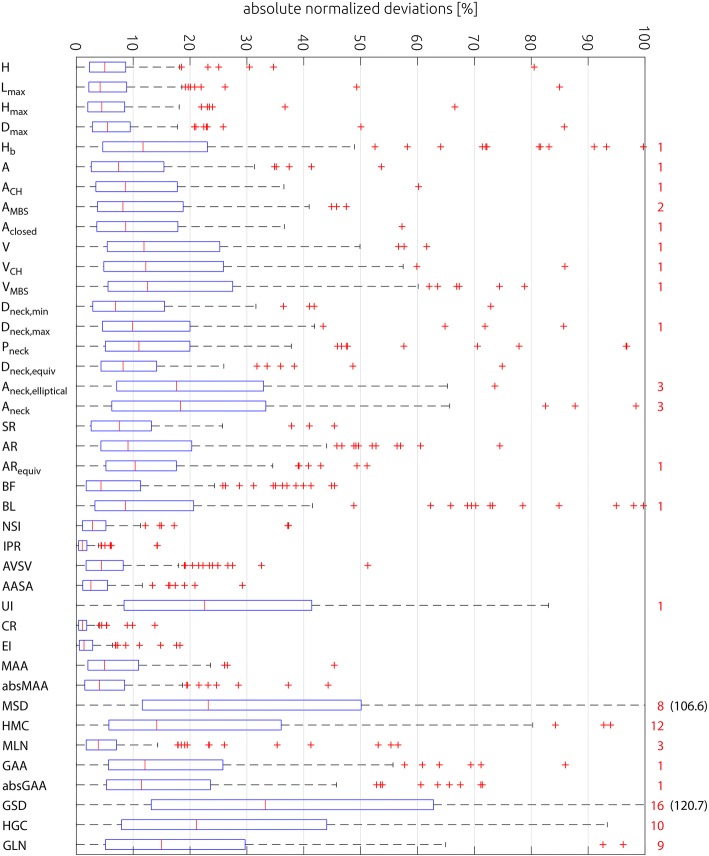
Boxplots of absolute relative deviations between measured values and the median of the respective aneurysm for all investigated geometric parameters. The plot was cropped at 100%. The number of outliers omitted due to cropping is specified on the right side of the boxplot (red numbers). For two parameters (MSD and GSD) the 75% percentile whisker is omitted as well and these values are specified in parentheses next to the plot

Furthermore, the absolute relative deviations were calculated for each parameter. Here, the absolute deviation between each parameter value and the median of the parameter for the respective aneurysm was calculated and subsequently normalized using the same median value. These absolute relative deviations (121 values) were then visualized using a boxplot for easier comparison of relative parameter uncertainties (see Fig. 1).

### Statistical analysis

Statistical analysis was performed using SPSS. All data was tested for normality using the Shapiro–Wilk test. Since a large subset of parameters was not normally distributed, we used median and IQR to describe central tendency and variability, respectively. Correlation between parameters was quantified using Kendall’s tau τ. To determine whether a parameter’s uncertainty is affected by the parameter’s average value, correlations were calculated between median values and relative uncertainties calculated for each parameter and aneurysm. Furthermore, correlations were calculated between all 120 parameter values to identify similar parameters. Here, correlations of parameters describing similar geometric aspects are reported for τ > 0.7. Additionally, Wilcoxon signed-rank test was performed on the absolute relative deviations to assess differences in relative uncertainties between parameters. The significance level of all tests was set at 0.05.

## Results

### Uncertainty of size parameters

Table 2 summarizes averaged values and relative uncertainties of 12 aneurysm size parameters calculated for the five investigated IAs.

**Table 2:** Evaluation of size parameters

#	Name (abbreviation)	Value, median [IQR]	Relative uncertainty, median [IQR] in %	Correlation, τ
1	Height (H)	2.94 [2.60] mm	14.0 [9.6]	1.0
2	Maximum dimension (L_max_)	5.13 [2.49] mm	15.7 [14.2]	0.4
3	Maximum height (H_max_)	3.52 [2.48] mm	12.3 [15.2]	0.6
4	Maximum diameter (D_max_)	4.90 [2.38] mm	16.9 [15.2]	0.4
5	Bulge height (H_b_)	1.79 [1.10] mm	37.3 [77.4]	0.8
6	Surface area (A)	44.80 [45.94] mm^2^	21.5 [19.3]	1.0
7	Convex hull surface area (A_CH_)	52.08 [49.26] mm^2^	27.7 [22.6]	1.0
8	Minimal bounding sphere surface area (A_MBS_)	83.41 [76.15] mm^2^	30.5 [29.1]	1.0
9	Closed surface area (A_closed_)	51.34 [48.97] mm^2^	28.3 [21.9]	1.0
10	Volume (V)	29.97 [45.93] mm^3^	39.2 [31.1]	1.0
11	Convex hull volume (V_CH_)	32.37 [47.30] mm^3^	39.9 [32.6]	1.0
12	Minimal bounding sphere volume (A_MBS_)	71.63 [97.28] mm^3^	45.1 [44.6]	1.0

Median and IQR of the five median values calculated for each aneurysm (2nd column) as well as the parameters’ relative uncertainty ranges (3rd column). A measure of correlation, Kendall’s tau, between median values and uncertainties is specified (4th column)

Among 1D size parameters, the relative uncertainty of H_b_ (#5) was considerably higher than the relative uncertainties of the other parameters. Ignoring H_b_, the relative uncertainties of 3D size parameters were higher than the relative uncertainties of 2D size parameters, whose relative uncertainties were in turn higher than the 1D size parameters’ relative uncertainties.

### Uncertainty of neck size parameters

Table 3 summarizes averaged values and relative uncertainties of six parameters (four 1D and two 2D) describing neck size. Relative uncertainties of all 1D neck size parameters were approximately twice as high as the relative uncertainties of 1D size parameters (#1–4), excluding H_b_ (#5). The relative uncertainties of both 2D neck size parameters were considerably higher than the relative uncertainties of the 1D neck size parameters.

**Table 3:** Evaluation of neck parameters

#	Name (abbreviation)	Value, median [IQR]	Relative uncertainty, median [IQR] in %	Correlation, τ
13	Minimum neck diameter (D_neck,min_)	2.75 [0.90] mm	30.6 [15.6]	0.2
14	Maximum neck diameter (D_neck,max_)	3.59 [1.60] mm	34.8 [16.6]	0.8
15	Neck perimeter (P_neck_)	11.83 [5.54] mm	37.4 [28.0]	0.6
16	Equivalent neck diameter (D_neck,equiv_)	5.10 [1.81] mm	27.6 [13.6]	0.4
17	Neck area (A_neck_)	9.47 [6.10] mm^2^	59.3 [30.5]	1.0
18	Elliptical neck area (A_neck,elliptical_)	8.13 [5.17] mm^2^	65.0 [21.4]	1.0

Median and IQR of the five median values calculated for each aneurysm (2nd column) as well as the parameters’ relative uncertainty ranges (3rd column). A measure of correlation, Kendall’s tau, between median values and uncertainties is specified (4th column)

**Fig. 2:**
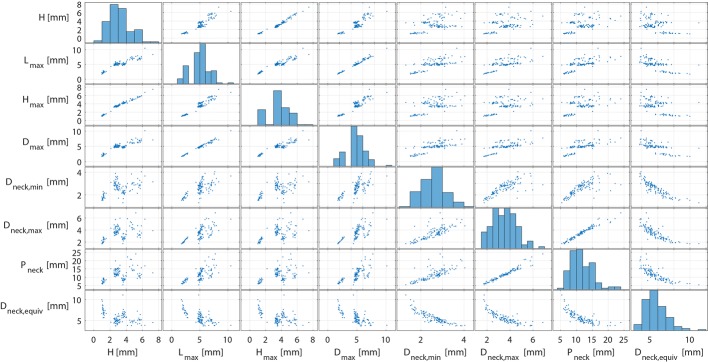
Scatter plot matrix of the four 1D size and the four 1D neck size parameters, which showed low uncertainties. Histograms of the respective parameters are shown along the diagonal entries

Figure 2 shows scatter plots between the four 1D size (excluding H_b_) and the four 1D neck size parameters (#13–16). No correlation with τ > 0.7 was found between 1D size and neck size parameters.

### Uncertainty of non-dimensional aneurysm shape parameters

Table 4 summarizes averaged values and relative uncertainties for twelve non-dimensional parameters aiming to describe aneurysm shape, deviation from a (hemi)spherical shape or aneurysm size relative to parent vessel size (#19).

**Table 4:** Evaluation of non-dimensional parameters

**#**	Name (abbreviation)	Value, median [IQR]	Relative uncertainty, median [IQR] in %	Correlation, τ
19	Size ratio (SR)	1.56 [0.88]	19.9 [14.5]	0.8
20	Aspect ratio (AR)	0.75 [0.64]	29.3 [28.1]	0.6
21	Equivalent aspect ratio (eAR)	0.57 [0.48]	32.6 [13.3]	1.0
22	Bottleneck factor (BF)	1.34 [0.57]	20.1 [30.0]	0.8
23	Bulge location (BL)	0.49 [0.35]	30.1 [73.4]	0.2
24	Nonsphericity index (NS)	0.27 [0.02]	10.8 [6.4]	0.2
25	Isoperimetric ratio (IPR)	5.28 [0.15]	3.9 [2.6]	0.2
26	Aneurysm volume to bounding sphere volume (AVSV)	0.44 [0.04]	17.3 [24.9]	0.4
27	Aneurysm surface area to bounding sphere area (AASA)	0.62 [0.04]	9.5 [12.3]	0.8
28	Undulation index (UI)	0.05 [0.03]	70.9 [73.8]	− 0.2
29	Convex ratio (CR)	0.95 [0.03]	4.2 [2.4]	0.2
30	Ellipticity index (EI)	0.25 [0.02]	5.1 [4.3]	0.2

Median and IQR of the five median values calculated for each aneurysm (2nd column) as well as the parameters’ relative uncertainty ranges (3rd column). A measure of correlation, Kendall’s tau, between median values and uncertainties is specified (4th column)

**Fig. 3:**
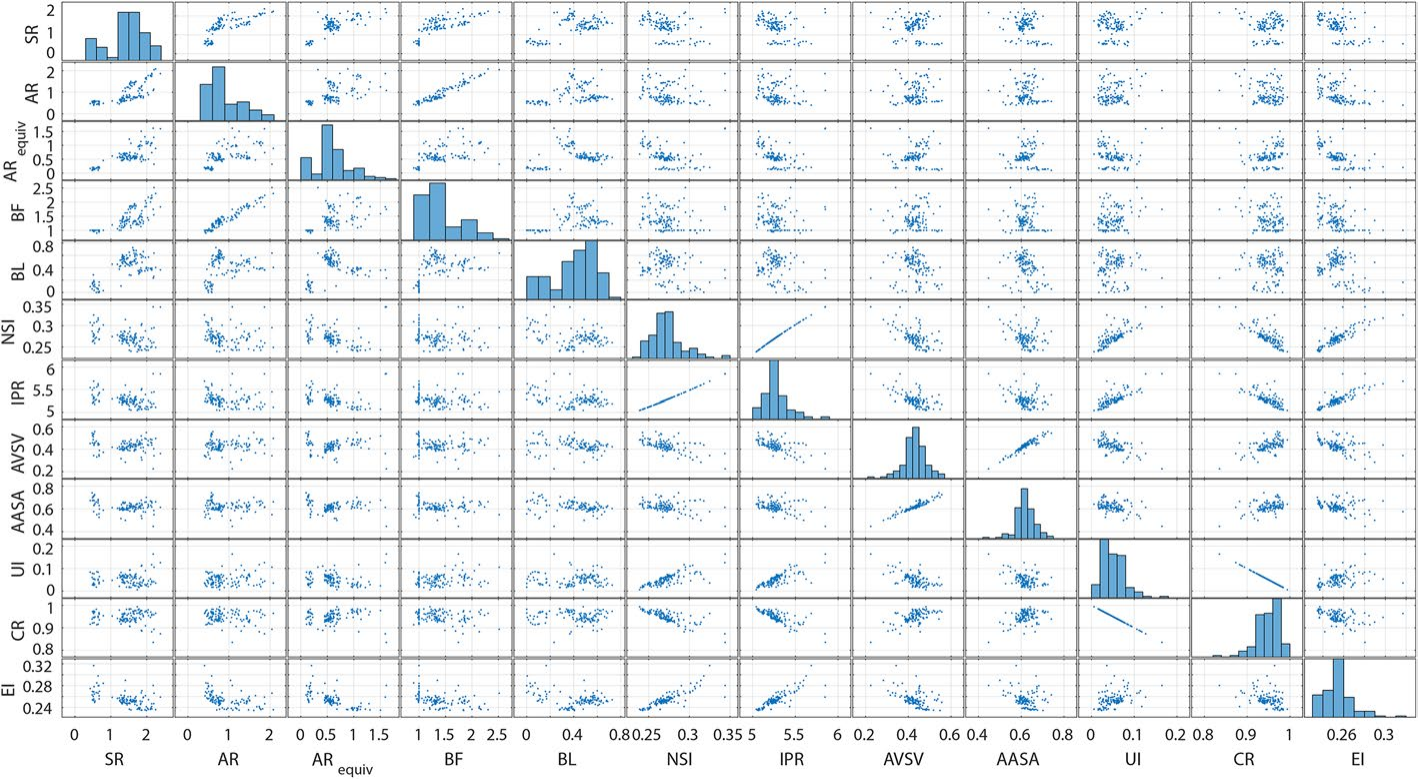
Scatter plot matrix of twelve non-dimensional shape parameters. Histograms of the respective parameters are shown along the diagonal entries

Figure 3 shows scatter plots for these twelve parameters. Four pairs of parameters seem to capture similar geometric aspects: AR and BF (#20, #22) correlate with τ = 0.80, NSI and IPR (#24, #25) correlate with τ = 1, AVSV and AASA (#26, #27) correlate with τ = 0.81, and UI and CR (#28, #29) correlate with τ = − 1 (since UI = 1-CR by definition). At the same time, relative uncertainty of BF is significantly lower than AR, relative uncertainty of IPR is significantly lower than NSI, relative uncertainty of AASA is significantly lower than AVSV, and relative uncertainty of CR is significantly lower than UI (all p < 0.001).

### Uncertainty of aneurysm surface curvature parameters

Table 5 summarizes averaged values and relative uncertainties for eight parameters characterizing aneurysm surface curvature.

**Table 5:** Evaluation of curvature parameters

#	Name (abbreviation)	Value, median [IQR]	Relative uncertainty, median [IQR] in %	Correlation, τ
31	Mean of mean curvature (MAA)	0.47 [0.25] mm^−1^	13.6 [15.6]	0.4
32	Mean of absolute mean curvature (absMAA)	0.50 [0.24] mm^−1^	13.3 [15.8]	0.2
33	Standard deviation of mean curvature (MSD)	0.11 [0.04] mm^−1^	101.1 [74.9]	0.0
34	High mean Curvature (HMC)	28.34 [18.72] %	39.6 [239.9]	0.0
35	L2-norm of mean curvature (MLN)	0.29 [0.07]	15.0 [19.0]	0.4
36	Mean of Gaussian curvature (GAA)	0.18 [0.23] mm^−2^	34.4 [45.6]	0.6
37	Mean of absolute Gaussian curvature (absGAA)	0.35 [0.35] mm^−2^	48.5 [42.7]	0.4
38	Standard deviation of Gaussian curvature (GSD)	0.24 [0.39] mm^−2^	179.8 [190.0]	0.4
39	High Gaussian curvature (HGC)	128.71 [71.35] %	82.2 [111.3]	0.6
40	L2-norm of Gaussian curvature (GLN)	1.86 [1.11]	54.5 [68.7]	0.8

Median and IQR of the five median values calculated for each aneurysm (2nd column) as well as the parameters’ relative uncertainty ranges (3rd column). A measure of correlation, Kendall’s tau, between median values and uncertainties is specified (4th column)

Two mean curvature parameters (#31 and #32) show a relatively low and similar relative uncertainty of around 15%. Both parameters are significantly correlated at τ = 0.8. They are also correlated with two 1D size parameters (#2 to #4) at τ > 0.7, as well as all 2D and 3D size parameters (#6 to #12) at τ > 0.8.

Among all other curvature parameters, only MLN (#35) has low relative uncertainty. However, only a weak correlation between MLN and the two mean curvature parameters mentioned above (#31 and #32) was observed (τ < 0.5).

## Discussion

Uncertainty associated with inter-operator or inter-group variability during segmentation was quantified for a large set of geometric parameters. A large variability in uncertainty for different parameters was found, with uncertainties ranging from 3.9 to 179.8%. This information is valuable since advanced image-based methodologies are increasingly applied to assess the rupture risk of individual IAs, aiming to support clinicians in making treatment decisions for individual patients. In these research efforts, several groups have attempted to develop a model predicting rupture risk of cerebral aneurysms based on geometric [12–16, 34] or hemodynamic parameters [30, 31], or a combination of both parameter types, as recently proposed by Detmer et al. [35]. However, the acceptance among physicians remains limited. One reason for this limited acceptance might be the difficulty in addressing and controlling all possible sources of error as well as often controversial findings of different research groups and differences in significance between univariate and multivariate analysis [16, 36]. Therefore, this study focuses on the quantification of uncertainty introduced during the crucial step of vessel wall reconstruction (segmentation and surface post processing, e.g. smoothing).

**Fig. 4:**
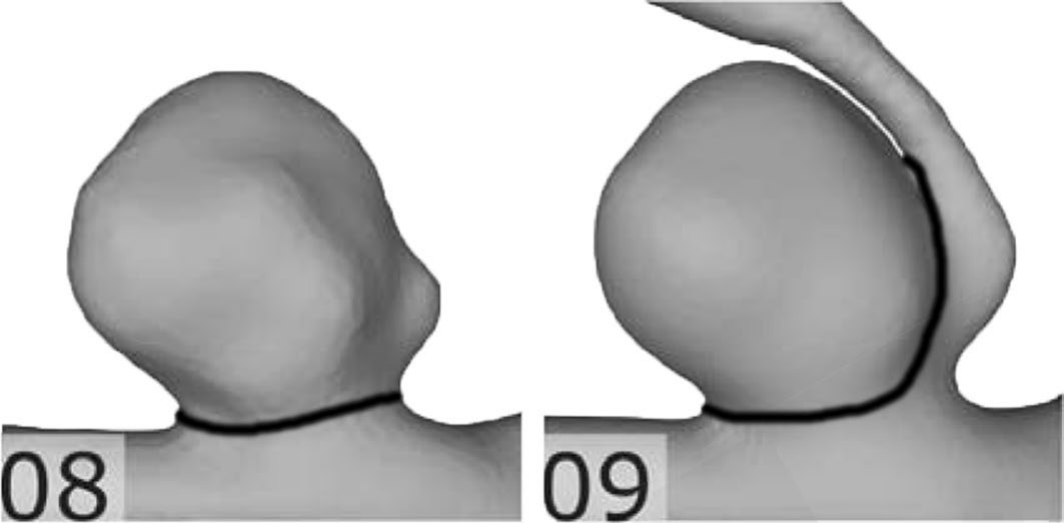
Example of differences in the aneurysm neck definition, depending on whether branching vessels were included in the image segmentation or not
- Curvature parameters generally exhibit the highest relative uncertainties, with the exception of three parameters, two of which (MAA and absMAA) are measures of aneurysm shape and size, while the third parameter (MLN) is a measure of aneurysm shape alone.

The uncertainties and their variability found in our study were caused by different segmentation and surface reconstruction approaches from medical image data. Considering the median values of the relative uncertainty ranges of all investigated parameters, the following key findings can be summarized:

- Non-dimensional shape parameters constructed from surface areas and/or volumes tended to exhibit the lowest uncertainties: IPR (#25), CR (#29), EI (#30). This is not surprising, since the parameters used for the calculation of these non-dimensional parameters are either overestimated or underestimated concordantly, thus reducing difference between non-dimensional values.
- Four out of five 1D size parameters also showed comparatively low relative uncertainty values: H, L_max_, H_max_, D_max_ (#1–4).
- Relative uncertainties of size parameters grow with dimension. Relative uncertainties for 2D size parameters (i.e. areas) were larger than for 1D size parameters and relative uncertainties for 3D size parameters (i.e. volumes) were larger than for 2D size parameters. Relative uncertainties for 2D and 3D size parameters were at least twice as high as for 1D size parameters. This increase in relative uncertainty with dimension seems reasonable, given that the 2D and 3D size parameters can be expected to correlate with the square and cube of the 1D size parameters and hence the same can be expected from the relative uncertainties. This result was anticipated, since a small earlier study of the reproducibility of aneurysm segmentation found a segmentation accuracy of around one voxel [25]. Thus, the relative uncertainty of a 1D size parameter can be approximated as the voxel size divided by the 1D parameter (e.g. diameter). Here, the voxel resolution of the image data was 0.28 mm and the average aneurysm diameter was 4 mm. Thus, an estimate for the expected relative uncertainty of 1D size parameters would be 7%. Since 2D and 3D size parameters correlated with the square and cube of 1D size parameters, respectively, the relative uncertainties can also be expected to be approximately the square/cube of the 1D size parameter uncertainties.
- Calculated uncertainties for 1D neck size parameters were approximately twice as high as for 1D size parameters. This can be explained by the aneurysm neck being particularly sensitive to segmentation uncertainty, as was shown by Berg et al. [22]. For example, different segmentation approaches can lead to the in-/exclusion of small branching vessels, which can considerably affect the neck shape and size. This may result in bifurcation aneurysms being classified as sidewall aneurysms, an example is shown in Fig. 4.

### Uncertainty analysis based recommendations

Based on the described findings, several recommendations can be formulated, which could be taken into account in related future studies:

- Non-dimensional parameters seem to be preferable, since over- or undersizing during segmentation is partially compensated by the normalization, which results in lower relative uncertainty.
- In a recent study by Liang et al. [18], which reviewed 46 morphologic and hemodynamic studies, aspect ratio was identified as the most relevant parameter for rupture risk assessment among geometric rupture risk parameters. However, our result suggest that the relative uncertainty of the aspect ratio is approximately 30%. Aneurysm height was another frequently used parameter, which exhibits a relative uncertainty of 14% in our study. Thus, processing of image data should be done particularly cautiously, if these parameters are to be evaluated.
- Most curvature parameters exhibit high relative uncertainties in our study. This likely results from the curvature being particularly affected by both major steps of the reconstruction procedure, segmentation and smoothing. From the viewpoint of uncertainty, the inclusion of curvature parameters, with the exception of MAA, absMAA, and MLN, for rupture risk estimation warrants caution. If curvature parameters are to be used, careful standardization of image reconstruction should be aimed for.
- In addition to relative uncertainty, the differences in parameter value between ruptured and unruptured aneurysms should also be considered, when choosing a rupture risk parameter. For example, Weir et al. found an average AR of 1.8 for unruptured and 3.4 for ruptured aneurysms [37], while we found a relative uncertainty of 29.3% for AR. This suggests that AR is able to discriminate between the two groups fairly well, even though it has only average relative uncertainty. On the other hand, Weir et al. also found an average maximum dimension of 7 mm for unruptured and 8 mm for ruptured aneurysms, while we found a relative uncertainty of 15.7% for the maximum dimension. Thus, the resulting absolute uncertainty can be expected to be similar to the difference between both groups, making the parameter less suited for discriminating between the two groups. The example shows that relative uncertainty alone is insufficient to assess whether a parameter is suited for rupture risk estimation.

### Limitations

The calculation of geometric parameters requires the separation of the aneurysm head from the parent vessel, which was done manually by one operator for all data sets. Although a small inter-operator dependency analysis found minor impact on the calculation of geometric parameters [31], this procedure could be replaced by an automated approach in the future [17].

Another aspect possibly affecting the results of the uncertainty analysis is the accuracy of the geometric parameters’ measurements. Beside the voxel resolution of the acquisition discussed above, the resolution of the surface meshes provided by MATCH participants was important. The average and standard deviation of the mesh resolution was 0.199 ± 0.096 mm with a range between 0.010 mm and 0.453 mm. Note, that the accuracy of the used measurement tools is much higher than both the mesh and voxel resolution, with at least 32 bit accuracy for mesh node coordinates. Since accumulation of numerical errors might reduce this accuracy, we performed a numerical experiment on a synthetic aneurysm, which had the shape of a spherical cap (diameter 5 mm, height 4 mm, triangular mesh resolution 0.2 mm). Diameter, surface and volume of the meshed synthetic aneurysm were calculated as usual and compared to the analytical solution, the differences were 0.016%, 0.17% and 0.31%, respectively. Hence, the uncertainty introduced by the measurement tools used in our study can be neglected.

Further limitations of this study are linked to the design of MATCH: The major limitation of this study is the small number of segmented IAs. This impedes comparison of inter-aneurysm and inter-operator variability, because the former can only be assessed on the basis of five aneurysms.

Additionally, MATCH only evaluated limited information on the reconstruction procedure used by each group. Examples of missing information are the segmentation thresholds as well as the surface smoothing algorithms used.

The present study investigated the uncertainty of individual geometric rupture risk parameters only. Uncertainty of complex rupture risk models including interactions between two or more geometric risk parameters, such as proposed by Prestigiacomo et al. [38] or Detmer et al. [39], for example, was not investigated. However, all dimensionless parameters investigated here are combinations of two single rupture risk parameters and all data necessary to assess the uncertainty of other complex geometric rupture risk models is provided. Finally, uncertainty of the reconstruction procedure also affects calculation of hemodynamic parameters proposed as rupture risk parameters, which are not part of this study. Note, that the majority of complex rupture risk models include both geometric and hemodynamic risk parameters. Within the MATCH challenge, 17 groups tried to predict ruptured aneurysm. Among these groups, 12 proposed models combining geometric and hemodynamic parameters, whereas 5 used only hemodynamic rupture risk predictors.

## Conclusions

Uncertainty analysis is mandatory on the road to clinical translation and use of rupture risk prediction models. The presented uncertainty analysis shows, that accurate rupture risk estimation requires reliable and reproducible assessment of geometric parameters. Thus, developing standards for reconstruction of aneurysm image data seems warranted. The data provided by this study might be able to help support development of future rupture risk prediction models by providing estimates of the uncertainties of individual parameters. In the future, an uncertainty analysis based on a larger aneurysm cohort, representing a more representative range of geometric rupture risk parameter values, is desirable.

## Additional file

**Additional file 1.** All geometric parameters calculated for the aneurysm geometries provided in frames of MATCH.
